# Preventive strategies in neuroimmunology

**DOI:** 10.1186/s42466-026-00490-8

**Published:** 2026-04-29

**Authors:** Karin Riemann-Lorenz, Anke Alberty, Maryam Balke, Anna Gorsler, Birte Elias-Hamp, Alex Maximilian Keller, Anke Lührs, Sina Cathérine Rosenkranz

**Affiliations:** 1 Institute of Neuroimmunology and Multiple Sclerosis, University Medical Center Hamburg-Eppendorf, Martinistraße 52, Hamburg, 20246 Germany; 2 Neuropraxis Medicentrum, Moenchengladbach, Germany; 3 Department of Early Neurological Rehabilitation, Cellitinnen Hospital St. Mary, Cologne, Germany; 4 Department of Rehabilitation Sciences, Faculty of Health, University of Witten/Herdecke, Witten, Germany; 5 Fachklinik für Neurologische Frührehabilitation, Kliniken Beelitz GmbH, Beelitz, Germany; 6 Faculty of Health Sciences Brandenburg, Brandenburg Medical School, Theodor Fontane, Brandenburg, Germany; 7 Neurological Private Practice, Hamburg, Germany; 8 Department of Neurology, Ameos Klinikum St. Clemens, Oberhausen, Germany

**Keywords:** Prevention, Lifestyle interventions, Physical activity, Exercise, Nutrition, Vitamin D, Smoking, Sleep, Stress, Rehabilitation

## Abstract

Neuroimmunological diseases arise from a complex interplay of genetic susceptibility, environmental exposures, and immune-mediated mechanisms. In this context, modifiable lifestyle and environmental factors may contribute to disease risk and disease course, at least in a subset of patients. Accordingly, while disease-modifying immunotherapy remains the cornerstone of treatment, lifestyle-related factors are increasingly recognized as relevant targets for prevention and supportive disease management. This review summarizes current evidence on physical activity, nutrition, substance use, sleep, stress, rehabilitation, and digital health interventions across all levels of prevention—primary to quaternary. Physical activity exerts immunomodulatory and potential neuroprotective effects through pathways involving cytokines, growth factors, cerebral perfusion, and immune cell redistribution, and is associated with improvements in symptoms, function, and quality of life. Nutritional factors, including obesity and vitamin D deficiency, may be associated with MS risk and disease progression, whereas evidence for specific diets or supplements remains limited. Smoking represents a well-established modifiable risk factor for both MS disease development and progression, while findings regarding alcohol consumption are inconsistent. Sleep disturbances and chronic stress could influence immune regulation and symptom burden and may act as both risk and disease-modifying factors. Rehabilitation constitutes a cornerstone of tertiary prevention, and emerging digital technologies, including tele-exercise and virtual reality, may offer promising low-threshold strategies to support long-term self-management. Overall, lifestyle and behavioral interventions represent powerful, supportive and complementary tools in neuroimmunology, that may enhance overall health and disease management. High-quality interventional studies are required to define the optimal role of preventive strategies alongside immunotherapy.

**Supplementary Information:**

The online version contains supplementary material available at 10.1186/s42466-026-00490-8.

## Background

Despite advances in pharmacotherapy, most neuroimmunological diseases, including multiple sclerosis (MS), remain chronic, highlighting the need for effective prevention. Preventive strategies may reduce disease risk before disease onset and, after manifestation, may modify early disease trajectories and slow progression, yet they remain underused in neuroimmunology. Growing evidence indicates that lifestyle and behavioral factors substantially influence immune regulation, neuroinflammation, and neuroplasticity and may therefore affect both disease susceptibility and long-term outcomes across all levels of prevention [74]. In addition, rehabilitation concepts and digital health technologies provide new opportunities to improve accessibility, adherence, and patient empowerment. However, despite increasing patient interest, the implementation of structured lifestyle interventions in routine neuroimmunological care remains inconsistent. This review summarizes the current evidence on modifiable lifestyle and behavioral factors in neuroimmunological diseases, with a particular focus on MS, and discusses implications for clinical practice and future research.

## Main text

### Physical activity

#### Primary prevention

Physical activity encompasses any skeletal muscle–driven movement that increases energy expenditure, whereas exercise refers to planned, structured, and repetitive activity aimed at improving or maintaining physical fitness [87]. Exercise likely influences numerous biological signalling pathways, including neurotrophins such as brain-derived neurotrophic factor (BDNF), vascular endothelial growth factor (VEGF), neurotoxic proteins, cerebral blood flow, myokines, cytokines, and changes in immune cell populations ([11, 28, 60, 61]; [ 70, 79, 115, 168]). Acute exercise induces rapid anti-inflammatory effects, partly mediated by muscle-derived interleukin-6 (IL-6), which — despite its classical proinflammatory role — triggers anti-inflammatory signalling via IL-1 receptor antagonist and IL-10 and suppresses tumor necrosis factor-α (TNF-α) production [116]. Of note, TNF-α is a key mediator of systemic inflammation, but it is important to note that exercise-induced modulation of TNF-α reflects general immunoregulatory effects and should not be equated with pharmacological TNF-α blockade, which has been associated with worsening of MS. These mechanisms may be especially protective in the early phases of autoimmune diseases of the CNS. Epidemiological data show that vigorous physical activity is inversely associated with MS risk [17, 30, 52, 167]. Animal studies support these observations: mice with prior voluntary wheel running before induction of experimental autoimmune encephalomyelitis (EAE), the standard animal model of MS, exhibit reduced susceptibility to clinical disease manifestation, delayed disease onset, and/or reduced disease severity, reflecting exercise-induced immunomodulatory effects [32, 72, 130, 134].

#### Secondary prevention

Through the aforementioned mechanisms, physical activity may also help delay or prevent the progression from an existing preclinical stage to clinically manifest disease. One epidemiological study showed that the initially observed reduction in MS incidence among physically active individuals was no longer evident after six years, suggesting that while regular moderate to high-intensity physical activity may not fundamentally prevent the onset of multiple sclerosis (MS) [30], it could delay the clinical manifestation and progression of the disease. Moreover, exercise is being discussed as a functional biomarker, measuring fitness performance and physical activity as parameters for monitoring disability progression or evaluating treatment efficacy.

#### Tertiary and quaternary prevention

Whereas exercise was formerly not recommended in people with MS (pwMS), reviews and meta-analyses have shown that there is no higher risk of relapses, adverse events, or adverse effects in pwMS when exercising. Exercise in pwMS improves strength and cardiorespiratory fitness and may alleviate neurological symptoms [78]. Meta-analyses and randomized controlled trials (RCTs) point to improvements in fatigue, walking capacity, balance, pain, depressive symptoms, and quality of life in people with MS [8, 22, 41, 48, 62, 104, 114, 118, 152, 157]. In contrast, the impact on cognition remains inconclusive [37]. Exercise is also increasingly recognized as a potentially disease-modifying intervention. However, this concept is largely supported by preclinical data. Although some data indicate a reduced relapse rate, a direct effect on disease activity, as inflammatory MRI findings, has not been conclusively established [119, 150]. Human studies in both healthy individuals and patients with neurological disorders indicate that regular exercise could confer structural and functional benefits to the central nervous system (CNS) [26, 33, 148, 161]. Studies point towards the fact that exercise has the potential to enhance overall functional connectivity in highly connected brain regions as well as in thalamocortical resting-state functional connectivity (RSFC) and address cognitive processing impairments, whereas effects on brain volume changes (measured by percent brain volume change, PBVC) and lesion volume typically seem to be unchanged during the intervention period [54, 124, 138, 148]. The strongest evidence exists for aerobic exercise, but beneficial effects have also been reported for high-intensity interval training, resistance and balance training, with combined endurance-strength programs appearing most effective [157]. Evidence for other neuroimmunological disorders remains scarce. A recent review of ten intervention studies involving 159 Myasthenia gravis patients shows improved muscle strength, which has a positive effect on daily functioning and quality of life [38]. Preliminary findings from small-scale studies in chronic inflammatory demyelinating polyneuropathy (CIDP) indicate that physical activity may help reduce symptom severity, enhance functional capacity, and improve quality of life [29, 90]. Altogether, the quality of evidence remains limited due to short study durations and methodological shortcomings [25]. In individuals with chronic neuroimmunological diseases who are often treated pharmacologically and rehabilitatively, exercise serves as a low-threshold, low-risk, and in comparison to pharmacological disease-modifying treatments, low-cost complementary intervention. It can reduce secondary complications of chronic immobility, help maintain autonomy, and counteract the need for passive care [31].

#### Conclusion and recommendation

Physical activity represents a multidimensional preventive strategy for individuals with neuroimmunological diseases since it exerts effects across all levels of prevention [20]. Nevertheless, individuals with neuroimmunological diseases generally exhibit lower physical fitness compared to the general population and also often experience reduced muscle strength [14, 107, 160, 162]. Evidence-based guidance suggests that healthcare providers should promote the safety and benefits of exercise and physical activity for pwMS. Recommendations should be individualized, taking into account comorbidities, functional impairment and patient preferences. For pwMS with EDSS 0–6.5, a combination of aerobic exercise and resistance training is suggested, e.g., at least 150 min of moderate-intensity or 75 min of vigorous-intensity exercise per week, combined with 2–3 resistance training sessions [63]. However, these recommendations are not yet adequately implemented in clinical practice. Limited awareness, disability-related barriers, and mobility restrictions hinder implementation; therefore, individualized, behaviorally supported training programs are essential and well tolerated even at higher intensities, with potential for sustained activity gains [144, 160]. Tele-exercise represents a promising approach to overcome access barriers and improve long-term outcomes, particularly in pwMS [106]. Overall, the evidence supporting the indirect functional benefits of exercise — such as improvements in muscle strength, endurance, mobility, and quality of life — is robust, whereas data on direct immunomodulatory or CNS-specific effects remain limited, highlighting the need for more translational studies to elucidate these mechanisms [132].

### Nutrition and supplements

#### Primary prevention

Direct evidence from prospective longitudinal studies on the influence of nutrition and supplement use on MS risk is scarce. A large US cohort study in women found no significant association between overall diet quality — assessed by indices such as the Alternate Mediterranean Diet (aMD) or Dietary Approaches to Stop Hypertension (DASH) index — and MS risk, noting that dietary patterns earlier in life might be more relevant [135]. In contrast, UK Biobank analyses suggested a non-significant protective trend for Mediterranean Diet (MD) adherence [7]. Case-control studies provide evidence that adherence to a Mediterranean-style diet or healthy dietary pattern is associated with a significantly reduced risk of developing MS [9, 108]. One hypothesis is, that this protective effect could be mediated through immunomodulatory and neuroprotective mechanisms of typical components of the MD, e.g. oleic acid, omega-3-poyunsaturated fatty acids (PUFAs), polyphenols and flavonoids, supporting beneficial alterations in the gut microbiota and a reduction in inflammation [47, 77]. Another study found that consuming fish/seafood at least once a week, or at least once a month with regular fish oil use, was associated with a 44% reduced odds of MS or Clinically Isolated Syndrome (CIS) [77]. Two other nutrition-associated factors contribute to MS disease risk: Vitamin D sufficiency and body weight. Epidemiological evidence highlights a latitude-dependent variation in MS incidence, suggesting that reduced UV radiation exposure and subsequent lower endogenous vitamin D synthesis may predispose individuals to MS [56]. Observational studies have consistently shown an inverse association between vitamin D status and the risk of developing MS [2, 89]. Recent Mendelian randomization studies provide additional support for a causal role of low vitamin D status in the development of MS [103, 126]. These studies also indicate that genetic factors influencing vitamin D levels are linked to MS risk. Obesity (BMI ≥ 30 kg/m²), often associated with unfavourable dietary habits, is consistently linked to an increased risk of MS or demyelinating events, particularly when present during childhood or adolescence [46, 52, 82, 105, 125]. Obesity drives chronic low-grade systemic inflammation through adipocyte-derived cytokines such as TNF-α or IL-6, providing a plausible biological mechanism [82, 125]. Additionally, obese individuals typically have lower circulating and bioavailable vitamin D, which may further contribute to increased MS susceptibility [52].

#### Secondary prevention

The avoidance of the aforementioned risk factors for MS may also delay or prevent the transition from CIS to manifest MS or reduce disease activity (e.g., relapses, number of lesions), but robust data are lacking. In a study with 334 CIS patients and a follow-up period of up to five years, Vitamin-D-levels ≥ 50 nmol/l were associated with a reduced conversion rate to clinical definite MS after 12 months, a reduced number of new active lesions and reduced change in brain volume [2]. A meta-analysis of prospective cohort studies found that an increment of 25 nmol/L in serum 25(OH)D levels was associated with an approximate 10% decrease in new relapses and a 14–31% reduction in the risk of new radiological inflammatory activity in patients with early relapsing MS [94]. In a secondary analysis of the BENEFIT trial, obese individuals were more likely to convert to MS and had a higher rate of relapse than individuals with normal weight [88].

#### Tertiary prevention

RCTs evaluating dietary or supplement effects on MS activity, symptoms, or progression generally suffer from methodological limitations (small samples, lack of blinding, short duration), resulting in low certainty of evidence. A network meta-analysis suggested that Paleolithic, low-fat, and Mediterranean diets may reduce fatigue and improve quality of life, but emphasized the low credibility of these findings [145]. Ketogenic diet, which is occasionally used in refractory status epilepticus [35], did not show effects on new T2 lesions [5]. For core disease outcomes such as relapse rates, MRI activity, and disability, RCTs of individual nutrients (e.g., biotin, polyunsaturated fatty acids) or antioxidants (e.g., lipoic acid, Ginkgo biloba) have largely shown no significant benefit [51, 101, 112, 158]. Thus, no specific diet or single supplement can currently be recommended for routine MS management. Observational studies provide more consistent associations. Higher adherence to a Mediterranean diet was linked to lower objective and patient-reported disability, better cognitive and motor performance, and reduced fatigue and depression in a cohort of 563 pwMS [65]. A 10-year prospective study (*n* = 223) found that a more pro-inflammatory diet was associated with an increased relapse risk, though not with EDSS progression [139]. Regular fish consumption was associated with a lower risk of confirmed disability worsening and progression to EDSS 3 and 4 in a large population-based cohort (*n* = 2719), particularly among individuals who maintained or increased intake post-diagnosis [59]. Body weight also appears relevant: obesity at MS onset (BMI ≥ 30 kg/m²) was associated with higher disability at baseline and after 2–6 years in the NationMS cohort [83], and with faster disability accumulation in Swedish registry data, including increased risk of cognitive worsening [169]. Notably, only obesity — not overweigh t— was linked to accelerated progression, suggesting a threshold effect [83]. Regarding vitamin D supplementation, RCTs are generally of low quality and provide inconsistent results, with meta-analyses finding no clear benefit for relapse rate or disability when used as add-on therapy [56, 89, 171]. The recent D-Lay trial reported reduced combined disease activity in CIS patients receiving vitamin D monotherapy, but no effect on relapses alone, disability, fatigue, or quality of life; benefits appeared greatest in individuals with severe vitamin D deficiency and normal BMI [155].

#### Quaternary prevention

PwMS show a high level of interest in dietary modification [137, 141], but first studies indicate low MS-specific nutrition knowledge and food literacy [128]. Many pwMS try to improve their diet after diagnosis; some of them avoid entire food groups or follow strict diets. These changes pose potential nutrition risks such as vitamin B12 deficiency or a reduced intake of omega-3-fatty acids and minerals such iron, iodine and zinc. Ultra-high vitamin D doses (e.g. > 50 000 IU/day for several weeks or months) are associated with toxic side effects, including hypercalcemia and kidney failure [56, 171]. Cases have been reported where uncontrolled intake of such doses led to persistent renal failure and severe hypercalcemia [34]. In more advanced stages of MS, mobility restrictions can make it impossible to maintain a healthy, balanced diet independently. The ability to shop for fresh food and prepare meals may be limited. Swallowing difficulties and digestive disorders can lead to an inadequate supply of vital nutrients. Incontinence may lead to restriction of water intake.

#### Conclusion and recommendation

**Fig. 1:**
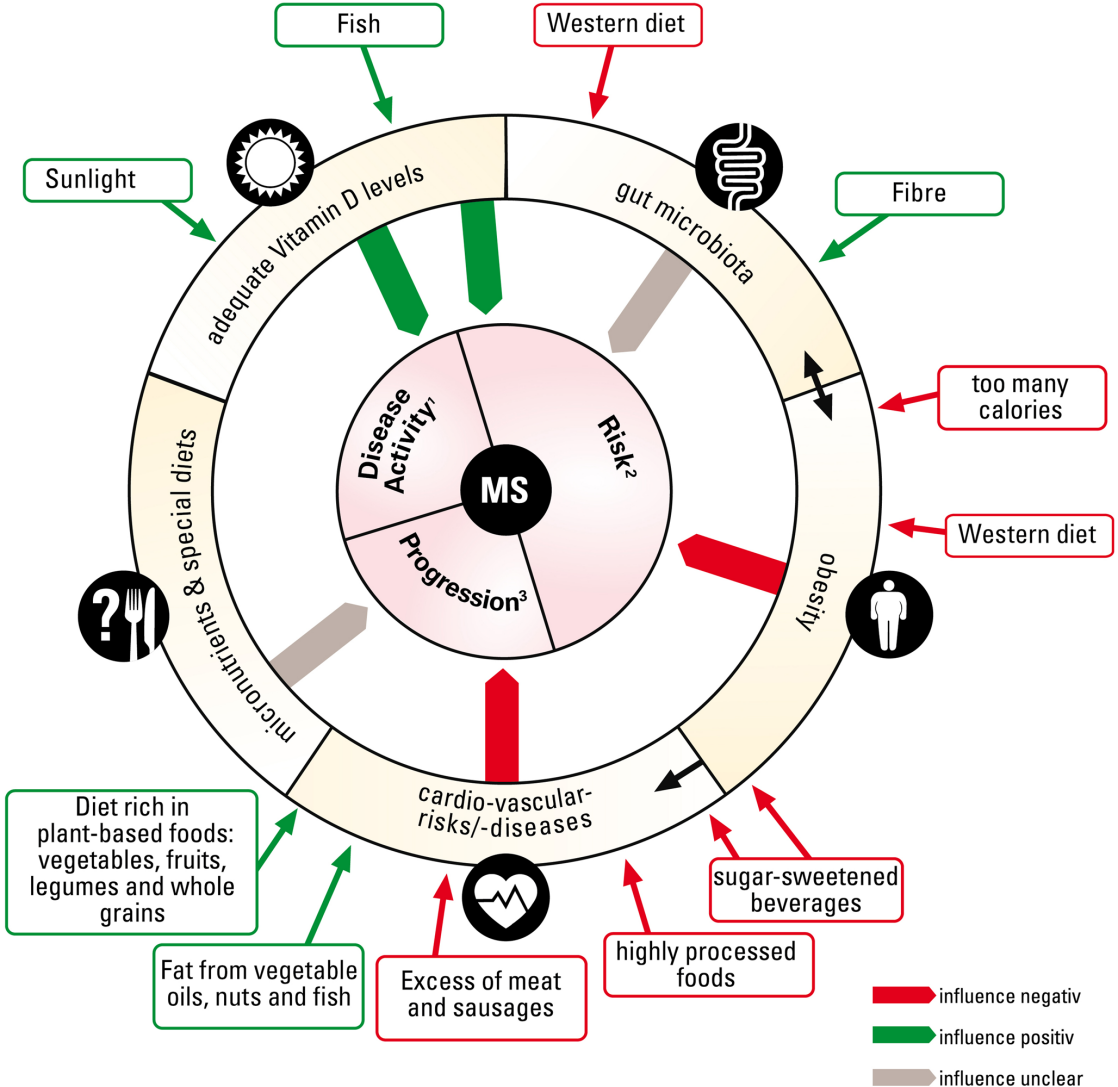
Graphical representation of the available evidence on diet and MS

Strong consistent evidence is lacking for dietary interventions in MS populations [145, 158]. However, studies increasingly provide evidence for the benefits of an overall healthy dietary pattern. Figure 1 depicts the available evidence on diet and MS. These benefits may stem from potential anti-inflammatory and neuroprotective mechanisms and a positive influence on gut microbiota exerted by ingredients predominantly found in plant-based foods. Moreover, healthy diets may reduce the risk for obesity, cardiovascular disease and diabetes [66], comorbidities associated with increased disability, and decreased quality of life in pwMS [93]. Hence, pwMS should be encouraged to follow a healthy dietary pattern as issued in national dietary guidelines [146]. Referral to professional nutritional advice should be considered when pwMS follow strict diets or face difficulties in implementing a healthy diet in everyday life. PwMS should be warned against excessively high vitamin D doses. For pwMS, emphasis remains on correcting vitamin D insufficiency rather than achieving ultra-high serum levels, given the disappointing results of vitamin D supplementation trials and the potential safety concerns associated with excessive supplementation. The tolerable upper intake level of 4000 IE/d by the US Institute of Medicine and 2000 IE/day by the European Food and Safety Authority (EFSA) should not be exceeded over longer periods of time [133]. Hence, persons with MS require trustworthy, straightforward, consistent, and MS-specific nutritional guidance, a need that is often neglected in standard MS care [127, 136]. Care from a specialized nutritionist and nursing support may be necessary in more advanced disease stages to prevent further deterioration of general health. Evidence for other neuroimmunological disorders is very scarce and preliminary findings [142, 156] or mechanistic considerations [40, 75] do not yet translate into dietary recommendations.

### Substance abuse (Smoking, Alcohol)

#### Primary prevention

Smoking is a well-established and significant modifiable risk factor for the development of MS [24]. Exposure to passive smoking is also linked to an increased risk of MS onset [140]. Even parental smoking at home has been associated with a doubled risk of childhood-onset MS [98]. Therefore, primary prevention strategies should strongly focus on smoking cessation within the general population, especially for individuals with a genetic predisposition or a family history of MS [98, 109]. In contrast, the evidence regarding the role of alcohol in MS onset is less definitive and somewhat contradictory, with some research suggesting it might be associated with a reduced risk of MS [45, 109, 165].

#### Secondary prevention

For individuals already diagnosed with MS, smoking is consistently associated with worse disease courses. This includes faster disease progression, increased disability, and adverse effects on long-term cognitive performance [24, 49, 50, 57, 93, 170]. Additionally, it is also associated with an increased risk for relapses and increased mortality in people with MS [10, 86]. Crucially, smoking cessation might reduce the risk of disease progression [151]. One study showed that different disease outcomes for those who quit smoking after diagnosis were not significantly different from those of non-smokers, highlighting the positive impact of cessation [96]. Regarding alcohol, studies indicate that alcohol dependence in pwMS is associated with an increased risk of anxiety and depression [96]. However, the direct effects of alcohol consumption on MS relapses and disability progression are inconsistent and require more robust research, as some studies found that moderate alcohol consumption was linked with no increased likelihood of disability, or with a decreased likelihood of disease progression [27, 165].

#### Tertiary and quaternary prevention

Despite the clear evidence of smoking’s harmful effects, the prevalence of unfavourable health behaviors, including smoking, remains high among pwMS [39, 73]. Many pwMS have limited awareness about the specific harms of smoking on MS progression. This lack of knowledge is compounded by a perceived ambivalence among pwMS regarding discussing smoking with their clinicians; they desire support but often feel shame or guilt [53, 68, 166]. Current smoking cessation support for pwMS is often inadequate. Clinicians frequently inquire about smoking status, but rarely offer evidence-based information or referrals to cessation services. Barriers for clinicians include limited consultation time, perceived lack of patient motivation, and insufficient availability of resources, such as information material [67].

#### Conclusion and recommendation

Smoking and alcohol consumption represent important modifiable lifestyle factors with potential impacts on MS development and progression. While smoking represents a well-established adverse factor across all stages of MS, the role of alcohol is less consistent. Addressing these modifiable risk factors is crucial for improving outcomes in pwMS. There is a need for improved knowledge dissemination, ensuring pwMS receive clearer education about smoking’s consequences [53, 68]. Empowering clinicians through training in evidence-based brief interventions and providing specific informational materials and referral pathways might be a promising approach [53, 67]. More robust studies are needed for smoking cessation interventions in pwMS, including research on e-cigarettes [57]. For alcohol, further observational studies with larger samples are required to explore the mechanisms of its effects on MS outcomes [165]. It is also important to acknowledge the methodological challenges in studies on lifestyle factors like smoking and drinking. A primary concern is residual confounding; for instance, smoking cessation often occurs in tandem with other health behaviours [159] (e.g., improved diet or increased physical activity), which may independently influence MS progression. While RCTs are ethically unfeasible for these exposures, future research should rely on high-quality prospective studies that utilize multivariable adjustment to better isolate the specific effects of smoking and alcohol from broader lifestyle shifts.

### Sleep

#### Primary prevention

Sleep is a vital physiological process that plays a key role not only in maintaining brain health but also in immune system regulation. Recent research has increasingly highlighted the link between sleep and the development or progression of neuroimmunological diseases. As these conditions involve complex interactions between the nervous and immune systems, sleep — being a key regulator of both — deserves our attention as a central factor in prevention strategies [95]. Research shows that chronic sleep deprivation disrupts immune homeostasis and leads to increased production of pro-inflammatory cytokines, IL-6 and TNF-α [55]. Given that neuroimmunological diseases are characterized by autoimmune inflammation of the central nervous system (CNS), it is plausible that sleep loss may increase vulnerability. Epidemiological data support this link. For instance, shift work, particularly during adolescence, has been associated with a significantly increased risk of developing MS [44]. Sleep is also tightly connected with the circadian system, and disruption of circadian rhythms may contribute to immune dysregulation, further compounding risk. Sleep deprivation also leads to an increased appetite, which may therefore lead to an enhanced consumption of foods promoting inflammation, such as red meats or highly processed foods. Improving sleep hygiene, reducing exposure to artificial light at night, and promoting consistent sleep-wake schedules could therefore be considered essential strategies in preventing the onset of neuroimmunological diseases.

#### Secondary prevention

In neuroimmunology, changes in sleep patterns may serve as early warning signs of emerging pathology. Sleep disturbances such as insomnia, restless sleep, REM sleep behaviour disorder, and excessive daytime sleepiness have been observed in patients before a formal diagnosis of conditions like MS [6]. These subtle changes may reflect early immune activation or brain inflammation, particularly in areas involved in sleep-wake regulation. Awareness of these contexts and active questioning of patients, as well as screening for sleep abnormalities in high-risk populations — such as individuals with a family history of autoimmune diseases or those with comorbidities like depression or obesity — might improve early identification of neuroimmunological disorders. This could facilitate timely treatment and prevent irreversible CNS damage. Moreover, sleep assessments are non-invasive and relatively low-cost, making them an attractive addition to early diagnostic protocols in neurology.

#### Tertiary prevention

Sleep plays a particularly important role, as sleep disturbances are highly prevalent among individuals with neuroimmunological disorders and can exacerbate core symptoms. In MS, for example, patients often suffer from chronic fatigue, cognitive impairment, mood disturbances, and pain —all of which are worsened by poor sleep quality. Studies show that targeted behavioural sleep interventions, such as Cognitive Behavioral Therapy for Insomnia (CBT-I), can lead to improvements in both sleep and daytime functioning [143]. Pharmacological strategies, such as melatonin supplementation, have also shown promise due to potential neuroprotective and anti-inflammatory properties [42]. Since melatonin secretion is often suppressed in patients exposed to excessive artificial light or with circadian disruption, supplementation may help realign sleep cycles and provide additional neuroimmune benefits. Moderate physical exercise, such as walking, cycling or slow running has a beneficial effect on sleep onset time.

#### Quaternary prevention

Poor sleep can mimic or worsen neurological symptoms; there is a risk that sleep-related complaints might be misinterpreted as disease progression or relapse. This can lead to overuse of immunosuppressive therapies or diagnostic procedures. For instance, fatigue, one of the most common complaints in MS, is often treated with stimulants or antidepressants without addressing the underlying sleep disruption. A more cautious approach that includes sleep evaluation may help to avoid misdiagnosis, reduce medication burden, and promote more holistic care [76]. Promoting awareness of sleep’s role in neurological health among healthcare providers can therefore reduce harm, improve patient outcomes, and contribute to more rational, patient-centered care.

#### Conclusion and recommendation

Sleep is an underappreciated but powerful tool in the prevention and management of neuroimmunological diseases. From its role in immune regulation to its ability to influence symptom severity and quality of life, sleep touches every stage of disease development. Addressing sleep disturbances proactively should be an integral part of neurological care and prevention strategies. By optimizing sleep in patients with neuroimmunological diseases, clinicians can help manage symptoms such as fatigue more effectively, reduce relapse rates, and improve quality of life.

### Stress

#### Primary prevention

Eustress refers to positive, activating stress that enhances motivation and performance and is perceived as manageable. Distress, in contrast, is negative stress that is experienced as overwhelming and can lead to exhaustion, reduced performance, or health problems over time. The key difference lies less in the stressor itself than in the individual’s appraisal and available coping resources. Chronic distress is a major risk factor and disease modulator in neuroimmunological disorders. Distress influences endocrine and immunological processes by activating the hypothalamic-pituitary-adrenal axis (HPA axis) and the autonomic nervous system. While acute distress responses can have short-term adaptive effects, long-term distress leads to immunological dysregulation and increased release of pro-inflammatory cytokines. Epidemiological studies show that distress-related disorders, such as post-traumatic stress disorder, are associated with an increased risk of developing autoimmune diseases [147]. Effective strategies include resilience training and cognitive stress management and mindfulness-based techniques, which lead to a reduction in cortisol levels and act as a ‘stress buffer’ [15]. Furthermore, lifestyle measures such as sleep hygiene and social support, which indirectly modulate the effects of stress, help. These approaches could potentially reduce susceptibility to neuroimmunological disease manifestations in the long term, but further studies are needed to confirm this.

#### Secondary prevention

It has been repeatedly shown that acute distressful events are associated with an increased risk of MS relapses [58, 102]. Interventions in the early stages of disease could therefore be promising for various (neurological) disorders, but appear to vary greatly from person to person. Cognitive behavioral therapy (CBT) has been shown to effectively reduce fatigue, depressive symptoms and stress levels. In RCTs Mindfulness-Based Stress Reduction programs significantly improved stress resilience, fatigue, and quality of life [12, 117]. Biofeedback techniques enable physiological self-regulation and complement psychological methods. These methods could therefore offer the potential to modulate the course of the disease favourably at an early stage; further systematic studies would be desirable for this purpose.

#### Tertiary prevention

In cases of chronic disease progression, stress management primarily aims to alleviate symptoms and psychosocial comorbidities. Psychosocial stress is associated with increased disease activity, risk of relapse and progression in MS [58, 102, 164]. Meta-analyses show that mindfulness-based approaches reduce stress, depression and anxiety in MS, thereby contributing to psychosocial stabilization [12]. Yoga and tai chi improve fatigue, quality of life and subjective stress perception through a combination of physical activity and relaxation exercises [18]. Peer support programs increase self-efficacy and social participation, which has a long-term stress-buffering effect [97]. Although direct effects on objective disease markers, such as relapse rate or MRI findings, have hardly been studied and the results are inconsistent, patients benefit significantly in terms of quality of life and functional ability.

#### Quaternary prevention

In advanced stages of the disease or in a palliative context, the focus is on reducing unnecessary stress and maintaining quality of life. Low-threshold measures – such as breathing exercises, meditation-based relaxation, music or art therapy – can reduce stress levels in the long term. It is also important to involve relatives in order to avoid social isolation and stabilize psychosocial care.

#### Conclusion and recommendation

Stress prevention is a central component of multimodal strategies for neuroimmunological diseases across all levels of prevention. It works by reducing psychosocial stress and strengthening neuroendocrine and immunological regulatory mechanisms. While the direct influence on disease activity has not yet been conclusively proven, there is clear evidence of improvements in stress symptoms, depression, fatigue and quality of life.

### Rehabilitation

Rehabilitation programs encompass all elements of the International Classification of Functioning, Disability, and Health (ICF), addressing the disease’s impact on progression, bodily functions like vision, sensation, vestibular or cerebellar deficits, weakness, impaired balance and posture, spasticity, fatigue and levels of activity and participation in social and work activities [16]. Rehabilitation for pwMS aims to address these areas using secondary and tertiary prevention, restoration, compensation, and adaptation to guide recovery efforts and enhance overall functioning and well-being [1]. It can be administered as individual or combined therapies in an outpatient setting, at outpatient rehabilitation clinics, or within inpatient facilities, followed by aftercare. Rehabilitation is a powerful, yet often underestimated tool in the management of fatigue, as it equips patients with the skills to integrate existing MS-fatigue recommendations into their daily routines. The interdisciplinary team — comprising physicians, psychologists, occupational and physical therapists, and sports therapists — can assist in developing strategies for daily energy tracking, pacing activities, and incorporating exercise and relaxation techniques into everyday life [43, 69]. This interaction and education during rehabilitation empower patients, fostering self-management and independence. Rehabilitation can also help in improving cognitive function, which can significantly improve their quality of life (QoL) and mental health. Given the lack of approved drugs to address cognitive deficits in MS, cognitive re-education (CR) emerges as a crucial intervention. Traditional cognitive therapy by structured cognitive exercises, aimed at enhancing specific cognitive domains such as memory and attention [36, 131]. Group-based therapies in rehabilitation settings provide social interaction and peer support [99, 110]. Computer-Assisted Programs using digital platforms to deliver exercises that can be personalized for different cognitive deficits. Music therapy has shown potential in engaging patients more fully and providing a stimulating environment for cognitive rehabilitation [84, 85]. Rehabilitation for pwMS should commence as early as possible after diagnosis and continue consistently throughout the disease’s progression [129, 154]. It should be goal oriented and specific, measurable, achievable, relevant and time-bound (SMART), focusing on existing impairments. Walking ability, speed, endurance and balance should be measured with and without walking aids using standardized assessment tools to evaluate the success [4]. In severely affected pwMS (EDSS > 6), intensive robot-assisted gait training should be used to maintain the walking ability [121]. For patients who are able to walk, regular therapeutically guided gait training supported by endurance (treadmill, ergometer) and strength training are recommended to enhance walking speed [149]. To improve balance and reduce the risk of falls, specific training of strength, balance and hippotherapy or Thai Chi can be helpful [13, 41, 71, 113, 153, 163]. Additionally, walking aids like canes, rolling walkers, ankle-foot-orthoses (AFOs), hip-flexion-assist-orthoses (HFAO), or functional electrical stimulators (FES) can ameliorate gait patterns [100].

### Use of new technologies in prevention

Health and medical apps are increasingly utilized in MS management, particularly due to the young age of diagnosis in PwMS [120]. In Germany, apps like these are regulated as Digital Health Applications (DiGAs) through the Federal Institute of Drugs and Medical Devices. ELEVIDA, a German DiGA, serves PwMS experiencing fatigue [122]. It offers digital neuro-rehabilitation, self-assessment tools, and strategies for managing fatigue, promoting self-awareness and autonomy. Other apps help to continuously assess MS-related symptoms, including fatigue (ElevateMS [123], allow patients to report symptoms like bladder dysfunction and fatigue (BRISA) [111], or educate users through sections on MS, behavior, and emotion (Energize). Fimo Health is a CE-marked digital therapeutic that supports people with chronic immunological diseases, including multiple sclerosis, in the management of fatigue [120]. The app combines symptom tracking, evidence-based educational modules, and integration of wearable data to promote self-management and patient empowerment. Virtual reality (VR) creates immersive environments for therapeutic purposes, characterized by interaction, immersion, and imagination. In contrast, augmented reality overlays digital information onto the real world. VR is feasible and safe, showing high satisfaction and engagement levels in PwMS. It enhances physical function through real-time feedback and multisensory experience, improving balance, gait, and is used in fatigue management [23, 64]. A recent Cochrane meta-analysis found that VR was superior to conventional training in upper limb function, balance and postural control, participation, and QoL. Yet, there are not enough robust study data for the superior effect of VR on cognitive function and fatigue. Exergaming interventions like the Wii console, combine exercise with gaming technology, and have demonstrated promising results in enhancing balance in individuals with MS in neurorehabilitation and telerehabilitation [3, 81].

### Limitations, research gaps and future directions

Research on preventive strategies largely relies on epidemiological studies – this applies to both general and neuroimmunology-specific prevention research. While these studies provide important insights, they can only demonstrate associations rather than causal relationships and are susceptible to residual confounding. Consequently, findings derived from observational studies must be interpreted with caution. While rigorous RCTs would be desirable to address these issues, in many instances they are unethical (e.g., smoking and alcohol consumption) or logistically unfeasible. Existing RCTs (e.g., in the area of nutrition and exercise) often have methodological limitations, including small sample sizes, short duration and follow-up periods, high risk of bias, and substantial heterogeneity in interventions and outcome measures, limiting the robustness and generalizability of their findings [19, 21, 145, 158]. Hence, adequately powered RCTs with longer follow-up should be funded and conducted to address these limitations where possible (e.g., effect of smoking cessation or weight loss on relapse rates and progression), while high-quality prospective cohort studies with rigorous multivariable adjustment should be prioritized where RCTs are not possible. Additionally, gender medicine represents a major research gap. Despite pronounced sex related differences in MS susceptibility, immune regulation, and disease progression, most lifestyle and preventive intervention studies do not stratify outcomes by sex or gender. Hormonal status, body composition, and behavioural factors may substantially modify the immunological response to preventive strategies, underscoring the need for sex- and gender-sensitive study designs. Similarly, immunosenescence is likely to critically influence the effectiveness of preventive interventions. Age-related immune remodelling alters both innate and adaptive immune responses, yet older individuals and patients with progressive disease stages are systematically underrepresented in interventional studies. This limits the applicability of current recommendations to an ageing MS population. Finally, preventive medicine in neuroimmunology should increasingly adopt interdisciplinary approaches, particularly for patients receiving long-term immunomodulatory therapies who frequently develop relevant comorbidities. Comorbid conditions have been shown to negatively affect MS outcomes [91, 92], yet structured preventive strategies integrating interdisciplinary approaches remain scarce. Addressing these gaps is essential to prevent further functional decline and to move forward in preventive care in neuroimmunology. Importantly, while modifiable lifestyle factors could support disease management, they are only complementary to, but cannot replace, evidence-based immunotherapy [80]. Patients and clinicians should therefore view lifestyle interventions as adjunctive strategies that enhance, rather than substitute, medical treatment.

## Supplementary Information

Supplementary Material 1

## Data Availability

Not applicable.
