# Supplementary material for: Preventive strategies in neuroimmunology
Source: Neurol Res Pract. 2026 Apr 29;8(1):33. doi: 10.1186/s42466-026-00490-8 (PMC13130587; doi:10.1186/s42466-026-00490-8)
Supplement: Supplementary file 1 — Supplementary Material 1 [file 42466_2026_490_MOESM1_ESM.docx]

Case report:

A 32-year-old woman with relapsing–remitting multiple sclerosis (EDSS 2.5) presents for lifestyle counselling. She has a persistent sensory spinal cord syndrome. Her medical history is notable for a predominantly Western dietary pattern, overweight body mass index, regular tobacco use, and known vitamin D deficiency. She previously engaged in regular horseback riding and swimming but has markedly reduced these activities in recent months, as she is uncertain whether horseback riding is still safe given her MS diagnosis.

Lifestyle recommendations:

1. Nutrition:
   Transition to a healthy dietary pattern (e.g. *Mediterranean diet)*  rich in fruits, vegetables, whole grains, fish, nuts, and unsaturated fats (e.g., olive oil); reduce red meat, sugar, and processed foods.
2. Exercise:
   Regular aerobic and resistance training (e.g., swimming, cycling, yoga) for at least 150 minutes per week, adapted to fatigue and neurological limitations. Horseback riding can continue if symptoms are stable, supporting balance and core stability.
3. Vitamin D Supplementation:
   Correct deficiency with regular monitoring of serum levels (target 20-50 ng/ml).
4. Smoking Cessation:
   Strongly advised, as smoking is associated with an accelerated MS progression.
5. Sleep Hygiene:
   Establish a stable sleep routine and optimize sleep quality to reduce fatigue and support cognitive function.
6. Rehabilitation & Physiotherapy:
   Regular participation in neurological rehab or physiotherapy to maintain mobility, endurance, and functional independence.
7. Cognitive Training:
   Incorporate targeted cognitive exercises (e.g., digital brain training programs, memory strategies) to support cognitive performance.
